# Age-Related Differences in the Neural Processing of Idioms: A Positive Perspective

**DOI:** 10.3389/fnagi.2022.865417

**Published:** 2022-05-25

**Authors:** Su-Ling Yeh, Shuo-Heng Li, Li Jingling, Joshua O. S. Goh, Yi-Ping Chao, Arthur C. Tsai

**Affiliations:** ^1^Department of Psychology, National Taiwan University, Taipei, Taiwan; ^2^Graduate Institute of Brain and Mind Sciences, National Taiwan University, Taipei, Taiwan; ^3^Neurobiology and Cognitive Science Center, National Taiwan University, Taipei, Taiwan; ^4^Center for Artificial Intelligence and Advanced Robotics, National Taiwan University, Taipei, Taiwan; ^5^Graduate Institute of Biomedical Sciences, China Medical University, Taichung, Taiwan; ^6^Taiwan International Graduate Program, Interdisciplinary Neuroscience, Academia Sinica, Taipei, Taiwan; ^7^Department of Computer Science and Information Engineering, Chang Gung University, Taoyuan, Taiwan; ^8^Institute of Statistical Science, Academia Sinica, Taipei, Taiwan

**Keywords:** idiom, positive aspects of aging, language, experience, functional brain reorgization

## Abstract

We examined whether older adults benefit from a larger mental-lexicon size and world knowledge to process idioms, one of few abilities that do not stop developing until later adulthood. Participants viewed four-character sequences presented one at a time that combined to form (1) frequent idioms, (2) infrequent idioms, (3) random sequences, or (4) perceptual controls, and judged whether the four-character sequence was an idiom. Compared to their younger counterparts, older adults had higher accuracy for frequent idioms and equivalent accuracy for infrequent idioms. Compared to random sequences, when processing frequent and infrequent idioms, older adults showed higher activations in brain regions related to sematic representation than younger adults, suggesting that older adults devoted more cognitive resources to processing idioms. Also, higher activations in the articulation-related brain regions indicate that older adults adopted the thinking-aloud strategy in the idiom judgment task. These results suggest re-organized neural computational involvement in older adults’ language representations due to life-long experiences. The current study provides evidence for the alternative view that aging may not necessarily be solely accompanied by decline.

## Introduction

Aging has been portrayed as a process of deterioration. Like any developmental process, aging involves various aspects (physiological, cognitive, language, perceptual, motivational, and emotional) of changes. Physical deterioration seems inevitable with increased age from a biological perspective. Previous studies have illustrated age-related decline, including slower reaction time (Williamson et al., 2009), loss of muscular strength (Charlier et al., 2016), and lower cardiovascular efficiency (Freiheit et al., 2010). Thus, unsurprisingly, aging is often associated with frailty, characterized by multiple physiological impairments and dependency (Ferrucci et al., 2006; Kuh, 2007; Weiss et al., 2010).

Even if solely examining aging from a behavioral perspective, it also appears as if older adults always perform “worse” than younger adults, since it has been shown that the abilities gained throughout the first quarter of our lives will only decline in the latter part of development, including sensation (Ulfhake et al., 2002), perception (Faubert, 2002), and cognition (Park et al., 2002). With such a negative societal view on aging, older adults may also acquire negative perspectives on their capabilities, which may prevent them from engaging in social and physical activities (Palacios et al., 2009) and living independently (Coudin and Alexopoulos, 2010). Indeed, many studies have demonstrated the detrimental effects of *ageism* on older adults’ mental and physical health (Bryant et al., 2016). Moreover, Palacios et al. (2009) also suggested that older adults with less stereotypical views of aging were more likely to be socially and physically engaged. Thus, by changing perspectives on aging, or at least an aspect of the aging process, the whole society may benefit from enhanced older adults’ overall well-being.

The current study aims to provide an alternative perspective and evidence that aging might not necessarily be solely accompanied by decay. Language is one of the functions that develop rapidly in childhood and remains relatively stable from adulthood to later life (Gaskell et al., 2009; Ramscar et al., 2013), especially in terms of lexicon size (Gaskell et al., 2009). However, some studies also suggest that language capacity becomes more limited with age due to sensory and cognitive decline [e.g., Burke and Shafto (2004)]. For instance, compared to younger adults, older adults produced fewer words, took a longer time, and made more errors when performing list generation tasks and object naming tasks (Goral, 2004; Goral et al., 2007). In addition, Chan and Poon (1999) have shown that older adults performed worse than younger adults in the category fluency task, suggesting an alternation of the semantic network in the process of aging (Tomer and Levin, 1993; Kozora and Cullum, 1995). A functional magnetic resonance imaging (fMRI) study also demonstrated that language lateralization toward the left hemisphere declined after age 25 (Szaflarski et al., 2006). Since language is a heavily lateralized function, such decline was interpreted as an explanation for less efficient communication in old age [but see Chen et al. (2019)].

However, older adults’ decline in language functions can be interpreted differently. A well-researched language-related limitation in later life is the failure to retrieve appropriate words during conversations, known as the *tip-of-the-tongue* phenomenon (Juncos-Rabadán et al., 2010). Such a phenomenon of knowing a term but cannot immediately retrieve it from memory has been observed consistently when people age (Salthouse and Mandell, 2013). Burke et al. (1991) proposed that this phenomenon is due to infrequent word use, which leads to weaker connections between lexical and phonological nodes. However, Ramscar et al. (2013) proposed an alternative explanation. They interpreted this finding that older adults performed poorer on the pair association learning task was due to their larger lexicon size and more prosperous life experience. They suggested that existing knowledge of words might prevent older adults from forming new connections with previously unassociated words, implying that the tip-of-the-tongue phenomenon may also result from older adults’ larger vocabulary size. Other studies have also supported the view of Ramscar and colleagues. For example, Bialystok and Luk (2012) found that older adults outperformed their younger peers on similar tasks due to their larger vocabulary size. Additionally, Fiez et al. (1999) also found that older adults know more rare words than younger adults. From this perspective, language-related knowledge increases with age rather than declines.

Here we examined whether and how older adults process idioms differently from younger adults from both behavioral and neural aspects. Idioms are commonly used in daily life, especially in Chinese, as they contain a rich cultural and historic background (Yang et al., 2016) with a stable structure (Liu et al., 2010), and their use is a form of language ability that we continue to acquire from adolescence through adulthood (Chan and Marinellie, 2008). As idioms work as large words or lexical units (Nippold et al., 2007; Conner et al., 2011), older adults should preserve the ability to process idioms with their stabilized or even increased lexicon size. Indeed, Hung and Nippold (2014) showed no age-related decline when tasked with explaining idioms, and 60–69 years old older adults reported greater familiarity and provided better explanations to idioms than 20–29 years old younger adults. Thus, the ability to process idioms varies between older and younger adults, depending on their familiarity with idioms. Despite that some studies have examined the effect of aging on idiom production [e.g., Qualls and Harris (2003) and Hung and Nippold (2014)], those studies might not be generalizable to the Chinese language, given that Chinese is relatively character-based processing, whereas, English idioms are relatively sentence-based. In addition, the Chinese writing system consists of characters taking up a square-shaped space with a nonlinear configuration varied in character structure (Yeh, 2000; Yeh and Li, 2002; Yeh et al., 2003), and the cognitive process underlying Chinese is different from that of English words (Tan et al., 2001). To date, no previous studies have investigated how aging might impact the processing of Chinese idioms, which is one of the novelties of the current study.

Chinese idioms require character processing that involves orthographic, phonological, and semantic processing (Wu et al., 2012). Using fMRI to directly examine which components might vary with age has several advantages to tackle how aging influences Chinese idiom processing. First, the task-based fMRI allows us to examine whether different neural substrates are in charge of the processing of idioms across different age groups, which can help us determine if older and younger adults recruit different brain areas in processing idioms; Second, fMRI allows us to investigate whether there is any re-organized neural processing in older adults’ brain, where some brain regions might show more robust activation for older adults and positively correlate with behavioral performance. In comparison, the same pattern might be absent in younger adults.

## Materials and Methods

### Participants

Since no previous studies have investigated age differences in processing Chinese idioms, we referenced a relevant study, Hung and Nippold (2014), to calculate the required sample size. In comparing familiarity with idioms across age groups, η^2^ = 0.29 was found. According to G*Power ver. 3 (Faul et al., 2007), 34 participants (17 participants in each group) were required to detect age differences with power = 0.95. We recruited nearly 60% more participants than needed to verify our results to be more conservative. Hence, 28 older adults [mean age (SD) = 67.0 (4.96) years, age range 59–82 years old, male/female 9/18; mean years of education (SD) = 14.9 (3.21) years] and 30 younger adults [mean age (SD) = 23.2 (3.23) years, age range 19–31 years old, male/female 14/16; mean years of education (SD) = 15.9 (1.86) years] were recruited. All were right-handed Taiwanese with normal or corrected-to-normal vision and had Montreal Cognitive Assessment [MoCA; Nasreddine et al. (2005)] scores above or equal to 26 points. All participants gave written informed consent before participating. One older adult was excluded because of his excessive head movement in translation (>3 mm) during fMRI scanning, making the total number of older adults 27. This study was approved by the Research Ethics Committee at the National Taiwan University (201611HS004).

### Stimuli and Procedure

**FIGURE 1:**
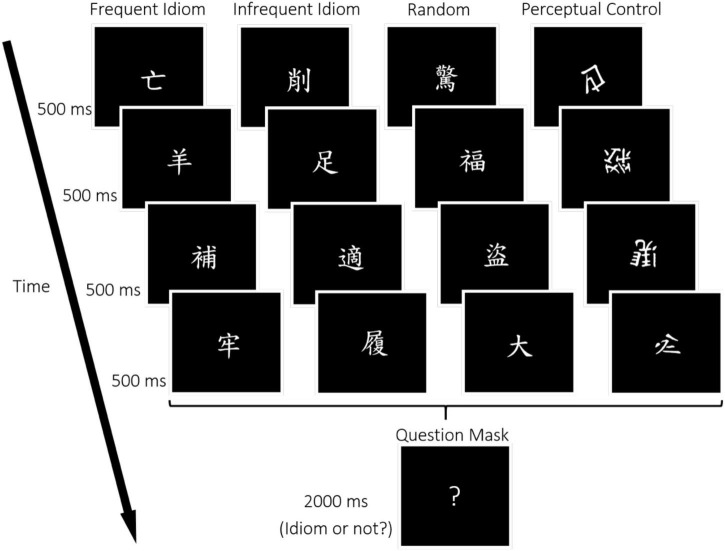
Sample experimental stimuli of the idiom judgment task. Four conditions were used for the target phase in this study, including Frequent Idiom (e.g., “亡羊補牢” is translated as “When the sheep run away, then you mend the sheepfold,” and means “To figure out a way to remedy the problem and prevent further loss”), Infrequent Idiom (e.g., “削足適履” is translated as “Cut off a piece of your feet to fit the size of the shoe” and means “To have inflexible thinking”), Random (character sequences with no meaning), and Perceptual Control (non-word sequences). For each trial, the four-character stimuli were presented sequentially after which a response display containing a question mark appeared, during which participants responded whether the quartets constituted an idiom or not.

Stimuli were presented using E-prime 2.0 (Psychology Software Toolbox, Sharpsburg, PA, United States) on a Windows PC. Participants viewed the stimuli *via* a head-mounted display in the scanner with 800 × 600 pixels resolution and 60 Hz refresh rate (Resonance Technology Inc., Northridge, CA, United States). Chinese idioms consisted of four characters (white, 4° × 4°) were presented serially one at a time at the center of a black background (Figure 1).

Four conditions were manipulated: frequent idiom, infrequent idiom, random, and perceptual control. Frequent and infrequent idioms were idioms of high and low frequency [mean (SD): 92.6 (49.66) vs. 6.84 (2.44) per million words; *t*_(24)_ = 8.88, *p* < 0.001], respectively. There were 25 frequent and 25 infrequent idioms adapted from the materials used in Zhou et al. (2016). In the random condition, non-idiom four-character Chinese strings were generated randomly, and these characters did not overlap with the characters of frequent and infrequent idioms. In the perceptual control condition, non-word character stimuli were created by rearranging Chinese characters’ strokes to form quartets of meaningless character-like stimuli. For the response cue display, a question mark (white, 4° × 2°) was presented at the same location as the Chinese characters.

There were 25 trials in each condition and 100 trials in total, equally distributed across five functional runs in this event-related fMRI experiment. There were five trials for each of the four conditions within a run, with the conditions presented in random order within each run. In each trial (Figure 1), during the target display, one of the characters of the four-character idiom was presented sequentially for 500 ms. After the quartet was completed, the response display with a question mark was presented for a maximum duration of 2,000 ms. Participants were instructed to judge whether the word sequence presented was an idiom or not during the response phase with assigned buttons as quickly and accurately as possible. After a response, the question mark would disappear, and a black background would appear until the entire 2000 ms period was over. A blank screen was then presented with the inter-trial interval (ITI) jittered between 3 and 4.5 s.

After the idiom judgment task, participants completed (1) the vocabulary test, the Wechsler Adult Intelligence Scale—Fourth Edition (WAIS-IV), and (2) the reading habits questionnaire based on Acheson et al. (2008). In the vocabulary test, 33 cards containing a Chinese two- or three-character word were shown to the participants, and they were asked to explain the meaning of each word. The reading habits questionnaire included author recognition and magazine recognition. Participants were asked to indicate from an author and magazine list the items they could recognize. The total score of these two tasks (author recognition and magazine recognition) indicates participants’ reading habits.

### fMRI Data Acquisition and Analysis

Scanning was conducted on a 3T Magnetom Prisma scanner (Siemens, Erlangen, Germany) with a 20-channel head coil at the Imaging Center for Integrated Body, Mind, and Culture Research of National Taiwan University, Taipei, Taiwan. For each participant, functional images were recorded using a gradient echo-planar imaging sequence with TR 2,000 ms, TE 32 ms, flip angle 87°, field of view 220 mm × 220 mm, voxel size 3.43 mm × 3.43 mm × 4.00 mm, 33 axial slices, and 390 scans. Axial slices were aligned parallel to the anterior-posterior commissural axis and placed for whole-brain coverage. We also acquired a T1-weighted Magnetization Prepared RApid Gradient Echo (MP-RAGE) sequence with TR 2,000 ms, TE 22.8 ms, flip angle 8°, field of view 256 mm × 256 mm, voxel size 1.00 mm isotropic, and 192 sagittal slices for registration and normalization to standardized template space.

Brain image data preprocessing and statistical analysis were performed using SPM12 (Wellcome Department of Imaging Neuroscience, London, United Kingdom). For each participant, functional volumes were realigned with unwarping to the first volume to correct for head motion and slice correction. Structural T1 images were then registered to the functional images and then segmented and normalized to the Montreal Neurological Institute (MNI) template space using the Diffeomorphic Anatomical Registration Through Exponentiated Lie algebra [DARTEL; Ashburner (2007)] procedure. T1 deformation parameters from the DARTEL procedure were then applied to the functional images with spatial smoothing using a 3D 8 mm Gaussian kernel.

First-level analysis of each participant’s fMRI data was conducted using a General Linear Model (GLM) with canonical hemodynamic functions. The GLM included four predictors corresponding to the four conditions (frequent idioms, infrequent idioms, random, and perceptual control) in the idiom judgment task, and the six head movements of each run were added to the model as regressors. Thus, first-level GLMs yielded whole-brain voxel-wise estimates of each participant’s neural responses to the four contrasts, including (1) idiom (frequent and infrequent idioms) vs. random, (2) frequent idiom vs. random, (3) infrequent idiom vs. random, and (4) idiom vs. perceptual control. These whole-brain neural response estimates were then submitted to second-level analysis.

Second-level analysis of the group-wise whole-brain neural response estimates was conducted using a two-sample t-test and multiple regressions to compare the contrast images of older and younger adults. The images were set at *p* < 0.001 (uncorrected) in all two-sample *t*-tests and multiple regression results. The cluster-wise correct False Discovery Rate (FDR) *q* < 0.05 was used in two-sample *t*-tests.

The Region Of Interest (ROI) mask was used in the multiple regression. The choice of ROIs was based on the overlapping brain regions between whole-brain neural results of idiom vs. random conditions and the semantically related areas (temporal and frontal regions) as suggested in the literature [e.g., Booth et al. (2002)]. The mask image of ROIs was produced by using XjView.^1^

## Results

### Behavioral Performance

Age differences in demographics, results of the vocabulary test score, and reading habits questionnaire are shown in Table 1. Two younger and two older participants did not complete the post-test tasks (vocabulary test and reading habits questionnaires) and thus were not included for further post-test analysis, but their data for other analyses were included. As shown in Table 1, there were no significant differences in gender [*x*^2^(1, *N* = 57) = 1.05, *p* = 0.306], education level [*t*_(55)_ = 1.47, *p* = 0.148], vocabulary test score [*t*_(51)_ = −0.02, *p* = 0.986], or reading habit score [*t*_(51)_ = −0.83, *p* = 0.411] for older and younger adults except for their difference in age [*t*_(55)_ = −39.88, *p* < 0.001].

**TABLE 1:** Basic demographics and reading skills of younger and older participants (Standard deviations in parentheses).

	Younger	Older	*p-*value
Gender (Male: Female)	14:16	9:18	0.306*^a^*
Age in years	23.2 (3.23)	67.00 (4.96)	<0.001*^b^*
Education in years	15.93 (1.86)	14.93 (3.21)	0.148*^b^*
Vocabulary test score	52.54 (5.04)	52.56 (4.96)	0.986*^b^*
Reading habit score	36.39 (11.48)	39.36 (14.52)	0.411*^b^*

*^a^Chi-square test. ^b^Two-sample t-test.*

**FIGURE 2:**
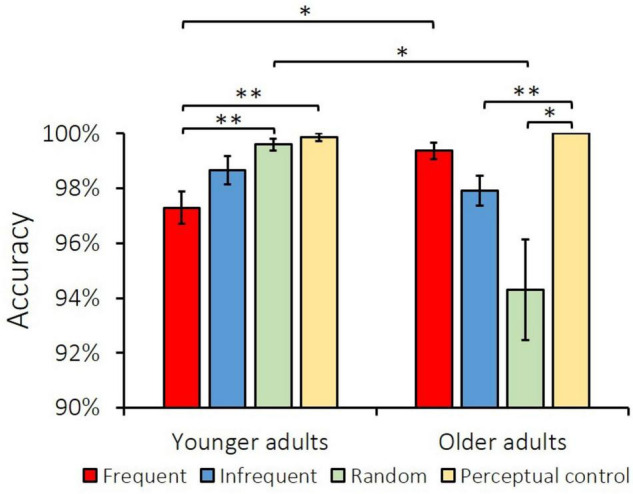
Accuracy (including hits for frequent and infrequent idioms, and correct rejection for random and perceptual control) for younger and older adults in the idiom judgment task across four conditions. Error bars represent one standard error of the mean. Note that the standard error of older adults’ perceptual control was zero. ^**^denotes *p* < 0.01, and *denotes *p* < 0.05.

Mean accuracies for the idiom judgment task are shown in Figure 2. Two types of trials were excluded from further analysis: trials in which participants responded before the response display and trials without responses. A two-way mixed repeated-measures analysis of variance (ANOVA) was applied on judgment responses with Age (younger and older) as the between-subjects variable and Condition (frequent, infrequent, random, and perceptual control) as the within-subjects variable. The ANOVA revealed a main effect of Condition [*F*_(3,165)_ = 6.04, *MSE* = 0.001, *p* = 0.001, *η_*p*_*^2^ = 0.099] but not Age [*F*_(1,55)_ = 3.43, *MSE* = 0.002, *p* = 0.069, *η_*p*_*^2^ = 0.059]. *Post-hoc* pairwise comparisons corrected using the Šidák method showed that accuracy was higher for the perceptual control (99.9%) than the frequent (98.3%) [*t*_(56)_ = 4.71, *p* < 0.001], infrequent (98.3%) [*t*_(56)_ = 4.18, *p* = 0.001], and random (97.1%) [*t*_(56)_ = 3.05, *p* = 0.007] conditions.

The interaction effect of Age x Condition was significant [*F*_(3,165)_ = 9.90 *MSE* = 0.001, *p* < 0.001, *η_*p*_*^2^ = 0.152]. Simple main effect tests corrected by the Šidák method showed that older adults had higher accuracy for perceptual control than the infrequent idioms [100.0 vs. 97.9%, *t*_(26)_ = 3.85, *p* = 0.004] and random [100.0 vs. 94.3%, *t*_(26)_ = 3.16, *p* = 0.024] conditions. In contrast, younger adults showed lower accuracy for frequent idioms than the perceptual control [97.3 vs. 99.9%, *t*_(29)_ = −4.56, *p* = 0.001] and random conditions [97.3 vs. 99.6%, *t*_(29)_ = −3.83, *p* = 0.004]. These results indicate that for both age groups perceptual control (scrambled characters) could be correctly judged compared to other conditions consisting of characters. However, older adults tended to misjudge random sequences as idioms more often than younger adults, which was further confirmed by the result that accuracy for the random condition was lower for older adults than younger adults [94.3 vs. 99.6%, *t*_(55)_ = −3.07, *p* = 0.012]. Most important and relevant to our hypothesis was the result that accuracy for frequent idioms was higher for older adults than younger adults [99.4 vs. 97.3%, *t*_(55)_ = 3.03, *p* = 0.016]. No other contrasts achieved statistical significance (*p*s < 0.05).

We also calculated individual *d’* scores for the recognition of frequent and infrequent idioms. The *d’* scores were acquired by subtracting the Z scores of the false alarm rates from those of the hit rates. The estimation of the *d’* scores is as follows:


(1) $$d^{\prime }\,Z_{A\,c\,c\,u\,r\,a\,c\,y_{i\,d\,i\,o\,m}}-Z_{\left(1-A\,c\,c\,u\,r\,a\,c\,y_{r\,a\,n\,d\,o\,m}\right)}\;$$

Correct identification of the frequent idioms was used as the hit rate in the frequent idiom condition, and that of the infrequent idioms was used as the hit rate in the infrequent idiom condition. These two conditions shared the same false alarm rate from the erroneous identification as idioms when they were random sequences in the random condition.

Two-sample *t*-test comparisons of the mean *d’* scores across age groups yielded no significant difference between older and younger adults in the frequent idiom condition [4.09 vs. 4.25, *t*_(55)_ = −1.17, *p* = 0.246] but significantly lower *d*’ for older than younger adults in the infrequent idiom condition [3.90 vs. 4.43, *t*_(55)_ = −3.44, *p* = 0.001]. In addition, we calculated individual β scores that indicate the bias to respond with one judgment more than the other. β larger than 1 indicates a tendency for “non-idiom” responses, whereas β smaller than 1 indicates a tendency for “idiom” responses. The calculation of β is as follows:


(2) $$\beta =\exp\left\{d^{\prime }\times -\frac{1}{2}\left[Z_{A\,c\,c\,u\,r\,a\,c\,y_{i\,d\,i\,o\,m}}+Z_{\left(1-A\,c\,c\,u\,r\,a\,c\,y_{r\,a\,n\,d\,o\,m}\right)}\right]\right\}$$

Kolmogorov-Smirnov test showed that the distribution of β is not normally distributed (all *p*s < 0.001), so the non-parametric test was used. Mann-Whitney *U*-Tests showed lower β for older relative to younger adults in the frequent idiom condition [*z* = 2.68, *p* = 0.007], but no age difference for β in the infrequent idiom condition [*z* = 1.65, *p* = 0.098] (Table 2).

**TABLE 2:** Mean *d’* and β estimates of response biases in older and younger adults.

	Index	Young	Older	*p-*value
Frequent	*d’*	4.25	4.09	0.246*^a^*
	β	2.25	1.34	0.007^b^**
Infrequent	*d’*	4.43	3.9	0.001^a^**
	β	1.77	1.47	0.098*^b^*

The calculations of the d’ and β are based on Equations (1, 2), respectively. ^a^Two-sample t-test. ^b^Mann-Whitney U-test. ** denotes p < 0.001.

For reaction time (RT) data, trials that were two standard deviations above the mean RT and RTs of incorrect responses were excluded from the analysis. As above, we applied a two-way mixed repeated-measures ANOVA with Age (younger and older) and Condition (frequent, infrequent, random, and perceptual control) as independent variables. This analysis yielded main effects of Age [*F*_(1,55)_ = 26.7, *MSE* = 42,061, *p* < 0.001, *η_*p*_*^2^ = 0.327], Condition [*F*_(3,165)_ = 31.0, *MSE* = 3,154, *p* < 0.001, *η_*p*_*^2^ = 0.361], and Age by Condition interaction [*F*_(3,165)_ = 3.51, *MSE* = 3,154, *p* = 0.017, *η_*p*_*^2^ = 0.060]. *Post-hoc* pairwise analyses corrected by using the Šidák method revealed faster RTs in younger than older adults (340 vs. 480 ms), and RTs of both frequent (372 ms) and infrequent (377 ms) conditions were faster than RTs of random (448 ms) and perceptual control (444 ms) conditions (all *p*s < 0.001). There were no differences between RTs for frequent and infrequent idioms, as well as random idioms and perceptual control non-words. For the interaction effect, younger adults had numerically faster RTs in the random condition than in the perceptual control condition [358 vs. 376 ms], and reversed result was found in older adults [539 vs. 512 ms]. However, the simple main effect test corrected by using the Šidák method could not obtain any statistically significant difference between the two conditions for either younger [*t*_(29)_ = 2.20, *p* = 0.195] or older adults [*t*_(26)_ = −0.92, *p* = 0.934].

### Neuroimaging Data

**FIGURE 3:**
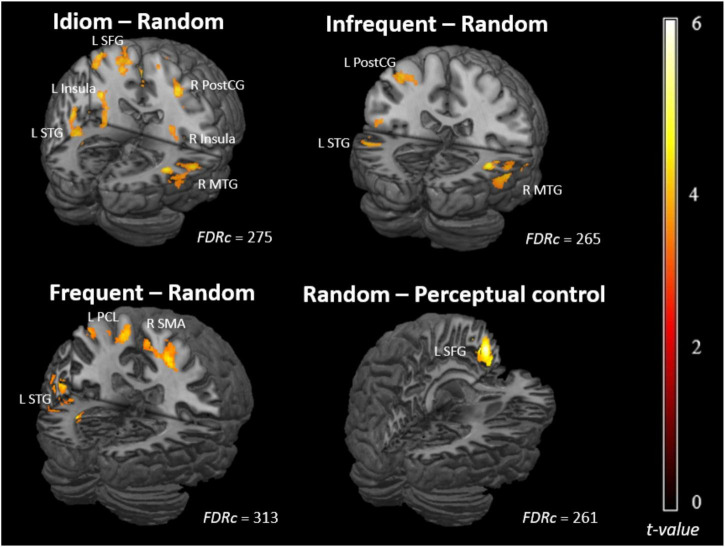
Whole-brain statistical contrast maps overlaid on 3D rendered template brains. Contrast maps depict brain areas in which the contrast responses for older adults were higher than those for younger adults. Voxel-wise statistical significance was set at cluster-wise corrected *q* < 0.05 False Discovery Rate (FDR). L, left; R, right; FDRc, FDR-corrected cluster threshold; SFG, superior frontal gyrus; STG, superior temporal gyrus; MTG, middle temporal gyrus; SMA, supplementary motor area; PostCG, postcentral gyrus; PCL, paracentral lobule.

We used whole-brain two-sample *t*-tests to evaluate brain areas in which older adults showed different contrast responses from younger adults across conditions (Figure 3 and Table 3). For idioms (frequent and infrequent included), relative to random characters, older adults engaged higher neural responses than younger adults in the left superior temporal gyrus (STG), bilateral insula, right postcentral gyrus (PostCG), left superior frontal gyrus (SFG), and right middle temporal gyrus (MTG). Considering the two types of idioms separately, for frequent idioms relative to random characters, older adults showed higher activation than younger adults in the right supplementary motor area (SMA), left STG, and left paracentral lobule (PCL). For infrequent idioms relative to random characters, older adults showed higher activation than younger adults in the right MTG, left PostCG, and left STG. In addition, for random characters relative to non-word characters (perceptual control), older adults engaged higher neural responses in the left SFG. However, no higher activations across conditions were found in older compared to younger adults.

**TABLE 3:** Peak activation details of brain areas with higher contrast responses in older than younger adults.

Brain region	# of voxels	q_*FDR–corr*_	X	Y	Z	*t*-value
**Idioms–random**						
L superior temporal gyrus	866	<0.001	−58	−12	−4	5.81
R insula	275	0.010	36	−2	16	5.79
L insula	301	0.008	−34	−8	16	4.42
R postcentral gyrus	484	0.001	44	−26	56	4.48
L superior frontal gyrus	1395	<0.001	−22	−2	60	4.65
R middle temporal gyrus	408	0.002	58	−40	6	4.19
**Frequent–random**						
R supplementary motor area	330	0.016	8	−24	60	3.89
L superior temporal gyrus	313	0.016	−58	−36	18	4.17
L paracentral lobule	527	0.002	−12	−20	64	4.17
**Infrequent–random**						
R middle temporal gyrus	458	0.002	58	−50	4	4.14
L postcentral gyrus	274	0.017	−42	−28	58	4.26
L superior temporal gyrus	265	0.017	−50	−36	22	4.05
**Random–perceptual control**						
L superior frontal gyrus	261	0.010	−6	50	28	5.40

*Whole-brain voxel-wise statistical significance was set at p < 0.001 (uncorrected) and cluster-wise corrected q < 0.05 False Discovery Rate (FDR). Coordinates of peak locations are in MNI space. L, Left; R, Right; # of voxels: Number of voxels in a cluster. q_FDR–corr_, cluster-level correct q-values using FDR.*

Based on the above whole-brain contrast of the idiom vs. random condition, ROIs were defined for the right MTG, left SFG, and left STG (see Materials and Methods). We then evaluated how ROI neural responses in younger and older adults were associated with behavioral performance during the idiom judgment task to see if there were compensatory activations or reorganization in older adults. Individual neural contrast response estimates were extracted from these ROIs and submitted to multiple regression analyses with *d*’ scores, which shows the behavioral performance of the idiom judgment task without the response bias in the frequent and infrequent conditions with age groups as predictors. The idiom words (frequent or infrequent) relative to random characters and frequent idioms relative to infrequent idioms are included as contrast pairs in the multiple comparison corrections to clarify how the compensatory/reorganization system works on the language function of word regularity (idiom words vs. random characters) and the frequency of idioms (frequent vs. infrequent). The reported areas of activation of the ROI analysis were significant using *p* < 0.05 Familywise Error (*FWE*) correction with the three predefined ROIs.

**FIGURE 4:**
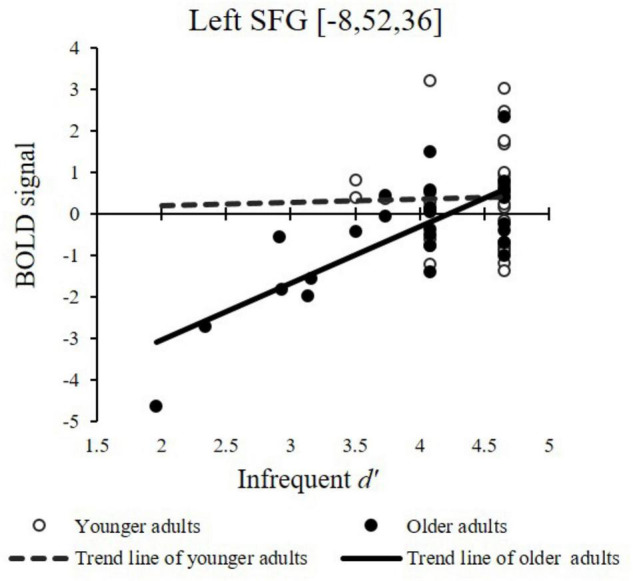
Associations between individual infrequent *d’* scores in the infrequent idiom vs. random condition and brain contrast responses in younger and older adults in the left Superior Frontal Gyrus showed significant whole-brain age difference in the neural-behavior correlation.

Region of interest analysis showed correlation trends in which greater neural responses to infrequent idiom than random conditions correlated with higher *d’* scores in older adults. Specifically, a positive correlation between the brain activation in the infrequent condition (contrasting with the random condition) and *d’* was found in the left SFG (*P*_*FWE–corr*_ = 0.011, voxel size = 97) in older adults (*r* = 0.747, *p* < 0.001; shown in Figure 4), whereas no such correlation was found in younger adults (*r* = 0.025, *p* = 0.895). We further confirmed this by applying a regression analysis using age and *d’* as independent variables to predict the brain activation in the left SFG. A significant interaction of age and *d’* was found, *t*_(53)_ = −2.11, *p* = 0.040, verifying the greater brain-behavior associations in the left SFG for older than younger adults (Figure 4).

## Discussion

This study showed differences between older and younger participants in behavioral and imaging data. We discuss the implications of these differences below.

### Behavioral Results of Idiom Processing

On the behavioral level, older adults showed higher accuracy for frequently seen idioms than younger adults. The results here thus indicate how experience benefits older adults, allowing them to be better than younger adults in making judgments on frequent idioms. No difference in the accuracy of infrequent idioms was found between older and younger adults, indicating the same level of performance in judging the less familiar idioms between the two age groups. Together, this supports our hypothesis that older adults preserve the language function in terms of idiom processing and can even perform better with frequent idioms than their younger counterparts.

However, by taking *d’* as the dependent variable, we found no age differences in the sensitivity of idiom judgment with frequent idioms and lower sensitivity for older adults with infrequent idioms. Closer scrutiny further indicates that the average *d’* for older adults (4.08 and 3.9 for frequent and infrequent idioms, respectively) was not low compared to the *d’* in younger adults (4.25 and 4.43 for frequent and infrequent idioms, respectively); this can further be verified by the similar accuracies (>97%) in infrequent idioms across age groups. It is to be noted that as the calculation of *d’* scores (of both frequent and infrequent idioms) involves the performance in random sequences (false alarm by mistaking these for idioms), how older and younger adults performed differently in this random condition is critical. Given that the *d’* measure depends on the responses to the random sequences which differed between older and younger adults, the accuracy of idiom judgment as mentioned above could better serve as a main behavioral proxy and the *d’* as an additional measure to reveal the results from different aspects.

It seems that older adults might not be able to correctly judge whether the random sequence was an idiom or not, as lower accuracy was found for older adults than younger ones. However, these results could also be due to older adults’ better world knowledge (Spreng and Turner, 2019), which requires a serial search through memory to accumulate more evidence to reject the random sequence as an idiom. Lee et al. (2018) have suggested that answering “no” is more challenging to assess and that answering “no” may require much more evidence in comparison to answering “yes.” Therefore, given older adults’ higher world knowledge, the more accumulated evidence is needed to form a rejection, which could have caused performance accuracy to be lower in the random sequence than in younger adults.

The finding of higher response bias (*β)* for younger adults in the idiom judgment for frequent idioms suggests that younger adults were more conservative in making judgments regarding frequent idioms. The result here also indicates the benefit that older adults acquire because of their more considerable world knowledge, enabling them to make more liberal judgments of the frequent idioms than younger adults. Yet, the age difference in bias was absent in the infrequent idiom condition, indicating that both age groups had the same criterion for judging infrequent idioms.

Despite the differences in Chinese and English writing systems, the current results align with Hung and Nippold (2014), who did not find age-related declines in idiom processing of English. On the other hand, Hung and Nippold (2014) found better performance in explaining idioms in individuals over 60 years older than younger adults in their twenties. Additionally, Qualls and Harris (2003) also found better performances in idiom interpretation in African-American older adults than younger adults when controlling for working memory capacity and reading comprehension ability. Therefore, the superiority or maintenance of language functions in idiom processing for older adults is not only applied to the English language system but can also be generalized to Chinese and different ethnicities, as shown in the current study.

### Higher Brain Activation in Older Adults Compared to Younger Adults

Compared to younger adults, older adults engaged in higher neural responses to idioms (both frequent and infrequent) relative to scrambled characters (i.e., the random condition) in the bilateral frontotemporal areas and the medial frontoparietal regions. This may indicate that older adults exert more cognitive resources in processing idioms than younger adults. For example, the SFG is in charge of working memory processing (Rypma et al., 1999). In addition, the STG is involved in the processing of tonal representations of Chinese characters (Tan et al., 2001) and is associated with the perception of intonation in speech prosody (Chang et al., 2010). The MTG is engaged in the extensive processing of spatial information in the Chinese language (Xiong et al., 2000) as a form of semantic representation (Booth et al., 2002, 2006). Higher activations in the insula and SMA were also found in older adults compared to younger adults. The activation in the SMA can be served as an index of motoric representation or articulatory rehearsal of vocally phonological information (Kuo et al., 2004) and the grapheme-to-phoneme conversion (Fiez et al., 1999). The insula has also been shown to mediate the motor aspect of speech production and articulatory control (Cereda et al., 2002; Oh et al., 2014). The higher activations in these brain regions suggest that older adults might recruit more resources to process idioms than younger adults. Older adults might also adopt the *thinking-aloud strategy* during the judgment of Chinese idioms as higher activations were found in several articulation-related areas, which might allow them to reduce cognitive load during the task (Smagorinsky, 1989). Together, we have shown that older adults adopted different strategies for judging Chinese idioms from a neuroimaging perspective.

We did not find higher activations in the fusiform gyrus (FG) and left inferior frontal gyrus (IFG) in older compared to younger adults. Both the FG and left IFG are highly associated with language processing, either in English or Chinese (Tan et al., 2005). In Chinese, the FG is related to the orthographic organization of Chinese (Bolger et al., 2005; Tan et al., 2005). As for the left IFG, previous studies have shown that the anterior and posterior part of the IFG is in charge of processing semantic and phonological information, respectively [see Wu et al. (2012) for review]. In addition, the IFG is associated with the processing of semantic integration in a sentence-level structure (Zhu et al., 2009). The absence of age differences in these two regions might indicate that older and younger adults recruited the same resources in processing orthographic organization and semantic integration across characters in the task. Although null results and inverse inference should be interpreted carefully, we consider this to be proper since the recognition of Chinese characters in skilled Chinese readers is based on orthographic constituents (Chen, 1996), which can easily be done by both younger and older adults in the current study. In addition, Atas et al. (2014) have shown that the temporal integration of symbols could be learned with trial-by-trial feedback. The learning of temporal regularity could even take place without visual awareness. Hence, with more experience in using idioms, older adults should have the ability to integrate temporal semantic information at the same level as younger adults.

When random vs. perceptual control conditions were compared, older participants recruited more high-level brain resources in the SFG than younger participants. No other brain regions showed significantly higher contrast responses in older than younger participants. These results are consistent with the behavior results: older adults made more effort during idiom processing, even with the random sequence characters. Moreover, a greater specific engagement of infrequent idioms relative to scrambled words across similar brain regions for semantic processing (left SFG) positively correlated with the task performance of older participants. These results suggest that when the brain regions correlated with idiom processing were active, older adults performed equally well as younger adults. Therefore, the activation in the SFG could be served as a form of compensatory mechanism (Cabeza et al., 2002), as the positive correlation between brain and performance was only found in older adults. Alternatively, given the higher accuracy of recognizing frequent idioms and higher brain activations for older adults than younger ones, this may not just be a form of compensation (which would imply a presence of a deficit) but a reorganization with behavioral advantages. Future studies that more specifically distinguish linguistic operations involved in processing idioms, which arguably are quite complex, are required to better evaluate the roles of the above brain regions as reflecting compensatory or re-organized neuro-computational involvement in older adult language representations.

### Current Views on Aging

By presenting the current study, we aim to combat the general stereotype regarding the decline of aging. While it is undeniable that certain capacity limitations are associated with the increase of age, there are potentially severe consequences when aging is evaluated solely from a negative perspective. With a view that aging is associated exclusively with deterioration, older adults may be perceived prejudicially and treated discriminatively even though most older adults do not experience frailty (McPherson, 2004). These negative beliefs from the public, the media, and policies seemed to influence older adults negatively. For example, age-related public policies, such as mandatory retirement age, can affect older adults’ sense of self and how others regard them (Hendricks, 2004). Many studies [e.g., Hausdorff et al. (1999); Chrisler et al. (2016), and Ng et al. (2016)] demonstrated that older individuals’ health and behaviors might be negatively affected in self-fulfilling ways as they embodied age-related stereotypes. Bai et al. (2016) also illustrated that those older adults with stronger beliefs about being a burden to their family have a higher risk of depression. Older individuals are affected physically and mentally by age-related negative stereotypes. They may require more social and healthcare support in a society where age is not respected, reinforcing the idea that older adults are weak or burdensome. This phenomenon was noted by Butler (2009), who coined the term *ageism* for negative stereotypes and discriminative acts against older adults. Older adults were greatly valued for their experience, knowledge, and institutional memory in the past. In Chinese culture, older adults have been regarded as the source of wisdom for their lifelong experiences. It has been considered virtuous for the younger generation to respect the older generation (Laidlaw et al., 2010).

### Future Directions

We used Chinese idioms as the stimuli to tackle the issue of age-related differences in language processing as idiom processing is similar to regular phrases or short sentences (Liu et al., 2010). The extraction of a regular or consistent pattern from the environment to expect upcoming stimuli occurs as early as infancy when infants learn to segment sentences into words (Saffran et al., 1996), and prior experience is essential for statistical regularity (Samuelson, 2002; Lany and Gómez, 2008; Salvagio et al., 2015). Chinese idioms have a very high transitional probability between characters and words (e.g., in the idiom “亡羊補牢”, the transitional probability between “亡羊” (“When the sheep run away”) and “補牢” (“then you mend the sheepfold”) is almost 100%). Such an ability to extract statistical regularity and expect upcoming events are crucial for daily lives. Future studies can examine other non-linguistic processing such as older adults’ motion perception and dynamic emotional expression to see whether similar re-organization or compensation also occurs in the aging brain albeit in different neural networks. That is, whether older adults, with rich experience and knowledge, would have better statistical regularity in general, or idiom processing is a special case in language processing to counter the aging brain.

In addition to reading, previous studies on idiom processing in speech [e.g., Liu et al. (2020)] revealed a dynamic network among temporal-parietal-frontal regions and the crosstalk between the dual-stream systems, whereby the ventral system is for comprehension and the dorsal system for articulation in speech (Hickok and Poeppel, 2007). Fei et al. (2020) found reduced activity of STG and IFG in older adults than in younger adults and increased frontal-temporal-parietal functional connectivity that may work to help facilitate idiom processing in older adults. It is worth comparing the difference between the two forms (visual vs. auditory) of idiom processing within the same experimental framework to find the commonality and differences, especially regarding the preservation of performance and the brain’s re-organization in older adults.

Finally, our finding that older adults could correctly judge frequent idioms better than younger adults and had the equivalent performance regarding infrequent idioms may be related to the expertise experience found previously in other domains. For example, experienced radiologists could recognize abnormal X-ray images better than novices, but not for normal X-ray images. Such a finding indicates that selective processing for distinguishable features increases with experience and this may hinder the detection of variations in normal images [Myles-Worsley et al. (1988), see also Evans et al. (2011)], an idea supported by eye-tracking results (Richter et al., 2020). It is likely that the older adults’ higher accuracy for frequent idioms reflects their expertise performance in language processing due to their lifelong experiences. Whether the two have shared underlying mechanisms awaits future studies.

## Conclusion

Compared to younger adults, we found better frequent-idiom recognition and higher neural activations in brain regions in charge of language processing and high-level functions for older adults. Older adults may benefit from experiences to help make judgments regarding Chinese idioms, which can be verified by their better (for frequent idioms) or similar performance (for infrequent idioms) in idiom judgments compared to younger adults. Despite previous studies investigating the deficits in semantic processing and reading in the aging population, this is the first study that directly examined the age effect on Chinese idiom processing with the measurement of neural correlates. This study provides another perspective on the aging process; namely, aging is not necessarily accompanied only by declines—instead, the experience accumulated across a lifespan with shifts in processing strategies.

## Data Availability Statement

The raw data supporting the conclusions of this article can be accessed on https://www.myqnapcloud.com/smartshare/608e947 k4l6p7075v536a699_9g6igg0lklnq2p11q5v25723593e4h2g.

## Ethics Statement

The studies involving human participants were reviewed and approved by the Research Ethic Committee at the National Taiwan University (201611HS004). The patients/participants provided their written informed consent to participate in this study.

## Author Contributions

S-LY: conceptualization, funding acquisition, and supervision. S-LY, JG, and S-HL: data curation. S-HL and JG: formal analysis. S-HL, JG, Y-PC, and S-LY: methodology. S-LY and S-HL: project administration and writing. S-LY, LJ, and AT: resources. S-HL: software. All authors: investigation and approved the submitted version.

## Conflict of Interest

The authors declare that the research was conducted in the absence of any commercial or financial relationships that could be construed as a potential conflict of interest.

## Publisher’s Note

All claims expressed in this article are solely those of the authors and do not necessarily represent those of their affiliated organizations, or those of the publisher, the editors and the reviewers. Any product that may be evaluated in this article, or claim that may be made by its manufacturer, is not guaranteed or endorsed by the publisher.

## Footnotes

1
www.alivelearn.net/xjview

## References

[B1] Acheson D. J. Wells J. B. MacDonald M. C. (2008). New and updated tests of print exposure and reading abilities in college students. *Behav. Res. Methods* 40 278–289. 10.3758/brm.40.1.278 18411551 PMC3022331

[B2] Ashburner J. (2007). A fast diffeomorphic image registration algorithm. *Neuroimage* 38 95–113. 17761438 10.1016/j.neuroimage.2007.07.007

[B3] Atas A. Faivre N. Timmermans B. Cleeremans A. Kouider S. (2014). Nonconscious learning from crowded sequences. *Psychol. Sci.* 25 113–119. 10.1177/0956797613499591 24186918

[B4] Bai X. Lai D. W. Guo A. (2016). Ageism and depression: perceptions of older people as a burden in China. *J. Soc. Issues* 72 26–46.

[B5] Bialystok E. Luk G. (2012). Receptive vocabulary differences in monolingual and bilingual adults. *Bilingual. Lang. Cogn.* 15 397–401. 10.1017/S1366728909990423 PMC4349351 25750580

[B6] Bolger D. J. Perfetti C. A. Schneider W. (2005). Cross-cultural effect on the brain revisited: universal structures plus writing system variation. *Human Brain Mapp.* 25 92–104. 10.1002/hbm.20124 15846818 PMC6871743

[B7] Booth J. R. Burman D. D. Meyer J. R. Gitelman D. R. Parrish T. B. Mesulam M. M. (2002). Modality independence of word comprehension. *Human Brain Mapp.* 16 251–261. 10.1002/hbm.10054 12112766 PMC6871904

[B8] Booth J. R. Lu D. Burman D. D. Chou T.-L. Jin Z. Peng D.-L. (2006). Specialization of phonological and semantic processing in Chinese word reading. *Brain Res.* 1071 197–207. 10.1016/j.brainres.2005.11.097 16427033 PMC2626184

[B9] Bryant C. Bei B. Gilson K.-M. Komiti A. Jackson H. Judd F. (2016). Antecedents of attitudes to aging: a study of the roles of personality and well-being. *Gerontologist* 56 256–265. 10.1093/geront/gnu041 24793646

[B10] Burke D. M. Shafto M. A. (2004). Aging and language production. *Curr. Direct. Psychol. Sci.* 13 21–24. 10.1111/j.0963-7214.2004.01301006.x 18414600 PMC2293308

[B11] Burke D. M. MacKay D. G. Worthley J. S. Wade E. (1991). On the tip of the tongue: what causes word finding failures in young and older adults? *J. Memory Lang.* 30 542–579. 10.1016/0749-596x(91)90026-g

[B12] Butler R. N. (2009). Combating ageism. *Int. Psychogeriat.* 21:211. 10.1017/S104161020800731X 18817584

[B13] Cabeza R. Anderson N. D. Locantore J. K. McIntosh A. R. (2002). Aging gracefully: compensatory brain activity in high-performing older adults. *Neuroimage* 17 1394–1402. 10.1006/nimg.2002.1280 12414279

[B14] Cereda C. Ghika J. Maeder P. Bogousslavsky J. (2002). Strokes restricted to the insular cortex. *Neurology* 59 1950–1955. 10.1212/01.wnl.0000038905.75660.bd 12499489

[B15] Chan A. S. Poon M. W. (1999). Performance of 7-to 95-year-old individuals in a Chinese version of the category fluency test. *J. Int. Neuropsychol. Soc.* 5 525–533. 10.1017/s135561779956606x 10561933

[B16] Chan Y.-L. Marinellie S. A. (2008). Definitions of idioms in preadolescents, adolescents, and adults. *J. Psychol. Res.* 37 1–20. 10.1007/s10936-007-9056-9 17592780

[B17] Chang E. F. Rieger J. W. Johnson K. Berger M. S. Barbaro N. M. Knight R. T. (2010). Categorical speech representation in human superior temporal gyrus. *Nat. Neurosci.* 13:1428. 10.1038/nn.2641 PMC2967728 20890293

[B18] Charlier R. Knaeps S. Mertens E. Van Roie E. Delecluse C. Lefevre J. (2016). Age-related decline in muscle mass and muscle function in Flemish Caucasians: a 10-year follow-up. *Age* 38:36. 10.1007/s11357-016-9900-7 26961694 PMC5005902

[B19] Chen P.-H. Wong J.-S. Lin W.-T. Tseng W.-Y. I. Goh J. O. S. Lee C. L. (2019). “Investigating the role of inter-hemispheric communication in age-related increase in right-hemisphere P600 grammaticality effect: a combined ERP and DTI study”. *Poster presented at the Eleventh Annual Meeting of the Society for the Neurobiology of Language.* Helsinki: SNL.

[B20] Chen Y.-P. (1996). What are the functional orthographic units in Chinese word recognition: the stroke or the stroke pattern? *Quart. J. Exp. Psychol. Sec. A* 49 1024–1043.

[B21] Chrisler J. C. Barney A. Palatino B. (2016). Ageism can be hazardous to women’s health: ageism, sexism, and stereotypes of older women in the healthcare system. *J. Soc. Issues* 72 86–104.

[B22] Conner P. S. Hyun J. O’Connor Wells B. Anema I. Goral M. Monereau-Merry M.-M. (2011). Age-related differences in idiom production in adulthood. *Clin. Linguist. Phonet.* 25 899–912. 10.3109/02699206.2011.584136 21728830 PMC3648420

[B23] Coudin G. Alexopoulos T. (2010). ‘Help me! I’m old!’How negative aging stereotypes create dependency among older adults. *Aging ment. Health* 14 516–523. 10.1080/13607861003713182 20480414

[B24] Evans K. K. Cohen M. A. Tambouret R. Horowitz T. Kreindel E. Wolfe J. M. (2011). Does visual expertise improve visual recognition memory? *Attent. Percep. Psychophys.* 73 30–35. 10.3758/s13414-010-0022-5 21258906 PMC3140200

[B25] Faubert J. (2002). Visual perception and aging. *Can. J. Exp. Psychol.* 56:164. 10.1037/h0087394 12271747

[B26] Faul F. Erdfelder E. Lang A.-G. Buchner A. (2007). G* Power 3: A flexible statistical power analysis program for the social, behavioral, and biomedical sciences. *Behav. Res. Methods* 39 175–191. 10.3758/bf03193146 17695343

[B27] Fei N. Ge J. Wang Y. Gao J.-H. (2020). Aging-related differences in the cortical network subserving intelligible speech. *Brain Lang.* 201:104713. 10.1016/j.bandl.2019.104713 31759299

[B28] Ferrucci L. Mahallati A. Simonsickm E. M. (2006). Frailty and the foolishness of Eos. *J. Gerontol. A Biol. Sci. Med. Sci.* 61, 260–261. 10.1093/gerona/61.3.260 16567374

[B29] Fiez J. A. Balota D. A. Raichle M. E. Petersen S. E. (1999). Effects of lexicality, frequency, and spelling-to-sound consistency on the functional anatomy of reading. *Neuron* 24 205–218. 10.1016/s0896-6273(00)80833-8 10677038

[B30] Freiheit E. A. Hogan D. B. Eliasziw M. Meekes M. F. Ghali W. A. Partlo L. A. (2010). Development of a frailty index for patients with coronary artery disease. *J. Am. Geriat. Soc.* 58 1526–1531. 10.1111/j.1532-5415.2010.02961.x 20633198

[B31] Gaskell M. G. Ellis A. W. (2009). Word learning and lexical development across the lifespan. *Philos. Trans. R. Soc. B Biol. Sci.* 364, 3607–3615. 10.1098/rstb.2009.0213 19933135 PMC2846319

[B32] Goral M. (2004). First-language decline in healthy aging: implications for attrition in bilingualism. *J. Neurol.* 17 31–52. 10.1016/s0911-6044(03)00052-6

[B33] Goral M. Spiro A. Albert M. L. Obler L. K. Connor L. T. (2007). Change in lexical retrieval skills in adulthood. *Ment. Lexic.* 2 215–238. 10.1093/cercor/bhu120 24907249 PMC4585486

[B34] Hausdorff J. M. Levy B. R. Wei J. Y. (1999). The power of ageism on physical function of older persons: reversibility of age-related gait changes. *J. Am. Geriat. Soc.* 47 1346–1349. 10.1111/j.1532-5415.1999.tb07437.x 10573445

[B35] Hendricks J. (2004). Public policies and old age identity. *J. Aging Stud.* 18 245–260. 10.1016/j.jaging.2004.03.007

[B36] Hickok G. Poeppel D. (2007). The cortical organization of speech processing. *Nat. Rev. Neurosci.* 8 393–402. 10.1038/nrn2113 17431404

[B37] Hung P.-F. Nippold M. A. (2014). Idiom understanding in adulthood: examining age-related differences. *Clin. Linguist. Phonet.* 28 208–221. 10.3109/02699206.2013.850117 24405225

[B38] Juncos-Rabadán O. Facal D. Rodríguez M. S. Pereiro A. X. (2010). Lexical knowledge and lexical retrieval in ageing: insights from a tip-of-the-tongue (TOT) study. *Lang. Cogn. Proc.* 25 1301–1334.

[B39] Kozora E. Cullum C. M. (1995). Generative naming in normal aging: total output and qualitative changes using phonemic and semantic constraints. *Clin. Neuropsychol.* 9 313–320. 10.1080/13854049508400495

[B40] Kuh D. (2007). A life course approach to healthy aging, frailty, and capability. *J. Gerontol. Ser. Biol. Sci. Med. Sci.* 62 717–721. 10.1093/gerona/62.7.717 17634317

[B41] Kuo W.-J. Yeh T.-C. Lee J.-R. Chen L.-F. Lee P.-L. Chen S.-S. (2004). Orthographic and phonological processing of Chinese characters: an fMRI study. *Neuroimage* 21 1721–1731. 10.1016/j.neuroimage.2003.12.007 15050593

[B42] Laidlaw K. Wang D. Coelho C. Power M. (2010). Attitudes to ageing and expectations for filial piety across Chinese and British cultures: a pilot exploratory evaluation. *Aging Ment. Health* 14 283–292. 10.1080/13607860903483060 20425647

[B43] Lany J. Gómez R. L. (2008). Twelve-month-old infants benefit from prior experience in statistical learning. *Psychol. Sci.* 19 1247–1252. 10.1111/j.1467-9280.2008.02233.x 19121132 PMC2967014

[B44] Lee A. L. Ruby E. Giles N. Lau H. (2018). Cross-domain association in metacognitive efficiency depends on first-order task types. *Front. Psychol.* 9:2464. 10.3389/fpsyg.2018.02464 30564183 PMC6288301

[B45] Liu Y. Li P. Shu H. Zhang Q. Chen L. (2010). Structure and meaning in Chinese: an ERP study of idioms. *J. Neurol.* 23 615–630.

[B46] Liu Z. Shu S. Lu L. Ge J. Gao J.-H. (2020). Spatiotemporal dynamics of predictive. brain mechanisms during speech processing: an MEG study. *Brain Lang.* 203:104755. 10.1016/j.bandl.2020.104755 32007671

[B47] McPherson B. D. (2004). *Aging as a social process: Canadian perspectives.* Don Mills: Oxford University Press.

[B48] Myles-Worsley M. Johnston W. A. Simons M. A. (1988). The influence of expertise on X-ray image processing. *J. Exp. Psychol. Learn. Memory Cogn.* 14 553–557. 10.1037//0278-7393.14.3.553 2969946

[B49] Nasreddine Z. S. Phillips N. A. Bédirian V. Charbonneau S. Whitehead V. Collin I. (2005). The montreal cognitive assessment, MoCA: a brief screening tool for mild cognitive impairment. *J. Am. Geriat. Soc.* 53 695–699. 10.1111/j.1532-5415.2005.53221.x 15817019

[B50] Ng R. Allore H. G. Monin J. K. Levy B. R. (2016). Retirement as meaningful: positive retirement stereotypes associated with longevity. *J. Soc. Issues* 72 69–85. 10.1111/josi.12156 27346893 PMC4920366

[B51] Nippold M. A. Mansfield T. C. Billow J. L. (2007). Peer conflict explanations in children, adolescents, and adults: examining the development of complex syntax. *Am. J. Speech Lang. Pathol.* 16 179–188. 10.1044/1058-0360(2007/022) 17456896

[B52] Oh A. Duerden E. G. Pang E. W. (2014). The role of the insula in speech and language processing. *Brain Lang.* 135 96–103. 10.1016/j.bandl.2014.06.003 25016092 PMC4885738

[B53] Palacios C. S. Torres M. T. Mena M. B. (2009). Negative aging stereotypes and their relation with psychosocial variables in the elderly population. *Arch. Gerontol. Geriat.* 48 385–390. 10.1016/j.archger.2008.03.007 18448179

[B54] Park D. C. Lautenschlager G. Hedden T. Davidson N. S. Smith A. D. Smith P. K. (2002). Models of visuospatial and verbal memory across the adult life span. *Psychol. Aging* 17:299. 12061414

[B55] Qualls C. D. Harris J. L. (2003). Age, working memory, figurative language type, and reading ability. *Am. J. Speech-Lang. Pathol.* 12 92–102. 10.1044/1058-0360(2003/055) 12680816

[B56] Ramscar M. Hendrix P. Love B. Baayen R. H. (2013). Learning is not decline: The mental lexicon as a window into cognition across the lifespan. *Ment. Lexic.* 8 450–481.

[B57] Richter J. Scheiter K. Eder T. F. Huettig F. Keutel C. (2020). How massed practice improves visual expertise in reading panoramic radiographs in dental students: an eye tracking study. *PLoS One* 15:e0243060. 10.1371/journal.pone.0243060 33270704 PMC7714201

[B58] Rypma B. Prabhakaran V. Desmond J. E. Glover G. H. Gabrieli J. D. (1999). Load-dependent roles of frontal brain regions in the maintenance of working memory. *Neuroimage* 9 216–226. 10.1006/nimg.1998.0404 9927550

[B59] Saffran J. R. Aslin R. N. Newport E. L. (1996). Statistical learning by 8-month-old infants. *Science* 274 1926–1928. 10.1126/science.274.5294.1926 8943209

[B60] Salthouse T. A. Mandell A. R. (2013). Do age-related increases in tip-of-the-tongue experiences signify episodic memory impairments? *Psychol. Sci.* 24 2489–2497. 10.1177/0956797613495881 24104505 PMC4291522

[B61] Salvagio E. Gomez R. Peterson M. (2015). Is prior experience necessary for 5.5 month-old infants to use the statistical regularity of an unchanging object on an changing background for segmentation? *J. Vision* 15:338.

[B62] Samuelson L. K. (2002). Statistical regularities in vocabulary guide language acquisition in connectionist models and 15-20-month-olds. *Dev. Psychol.* 38:1016. 10.1037//0012-1649.38.6.1016 12428712

[B63] Smagorinsky P. (1989). The reliability and validity of protocol analysis. *Writ. Commun.* 6 463–479.

[B64] Spreng R. N. Turner G. R. (2019). The shifting architecture of cognition and brain function in older adulthood. *Persp. Psychol. Sci.* 14 523–542. 10.1177/1745691619827511 31013206

[B65] Szaflarski J. P. Holland S. K. Schmithorst V. J. Byars A. W. (2006). fMRI study of language lateralization in children and adults. *Human Brain Mapp.* 27 202–212. 10.1002/hbm.20177 16035047 PMC1464420

[B66] Tan L. H. Laird A. R. Li K. Fox P. T. (2005). Neuroanatomical correlates of phonological processing of Chinese characters and alphabetic words: a meta-analysis. *Human Brain Mapp.* 25 83–91. 10.1002/hbm.20134 15846817 PMC6871734

[B67] Tan L. H. Liu H.-L. Perfetti C. A. Spinks J. A. Fox P. T. Gao J.-H. (2001). The neural system underlying Chinese logograph reading. *Neuroimage* 13 836–846. 10.1006/nimg.2001.0749 11304080

[B68] Tomer R. Levin B. E. (1993). Differential effects of aging on two verbal fluency tasks. *Percep. Motor Skills* 76 465–466. 10.2466/pms.1993.76.2.465 8483658

[B69] Ulfhake B. Bergman E. Fundin B. (2002). Impairment of peripheral sensory innervation in senescence. *Auton. Neurosci.* 96 43–49. 10.1016/s1566-0702(01)00368-x 11911501

[B70] Weiss C. O. Hoenig H. H. Varadhan R. Simonsick E. M. Fried L. P. (2010). Relationships of cardiac, pulmonary, and muscle reserves and frailty to exercise capacity in older women. *J. Gerontol. Ser. A Biomed. Sci. Med. Sci.* 65 287–294. 10.1093/gerona/glp147 19822621 PMC2822279

[B71] Williamson J. D. Espeland M. Kritchevsky S. B. Newman A. B. King A. C. Pahor M. (2009). Changes in cognitive function in a randomized trial of physical activity: results of the lifestyle interventions and independence for elders pilot study. *J. Gerontol. Ser. A Biomed. Sci. Med. Sci.* 64 688–694. 10.1093/gerona/glp014 19244157 PMC2679423

[B72] Wu C.-Y. Ho M.-H. R. Chen S.-H. A. (2012). A meta-analysis of fMRI studies on Chinese orthographic, phonological, and semantic processing. *Neuroimage* 63 381–391. 10.1016/j.neuroimage.2012.06.047 22759996

[B73] Xiong J. Rao S. Jerabek P. Zamarripa F. Woldorff M. Lancaster J. (2000). Intersubject variability in cortical activations during a complex language task. *Neuroimage* 12 326–339. 10.1006/nimg.2000.0621 10944415

[B74] Yang J. Li P. Fang X. Shu H. Liu Y. Chen L. (2016). Hemispheric involvement in the processing of Chinese idioms: an fMRI study. *Neuropsychologia* 87 12–24. 10.1016/j.neuropsychologia.2016.04.029 27143223

[B75] Yeh S. L. (2000). Structure detection of Chinese characters: Visual search slope as an index of similarity between different-structured characters. *Chin. J. Psychol.* 42 191–216.

[B76] Yeh S. L. Li J. L. (2002). Role of structure and component in judgments of visual similarity of Chinese characters. *J. Exp. Psychol. Human Percep. Perform.* 28:933. 10.1037/0096-1523.28.4.933 12190259

[B77] Yeh S. L. Li J. L. Takeuchi T. Sun V. Liu W. R. (2003). The role of learning experience on the perceptual organization of Chinese characters. *Visual Cogn.* 10 729–764.

[B78] Zhou J. Lee C.-L. Li K.-A. Tien Y.-H. Yeh S.-L. (2016). Does temporal integration occur for unrecognizable words in visual crowding? *PLoS One* 11:e0149355. 10.1371/journal.pone.0149355 26890366 PMC4758582

[B79] Zhu Z. Zhang J. X. Wang S. Xiao Z. Huang J. Chen H.-C. (2009). Involvement of left inferior frontal gyrus in sentence-level semantic integration. *Neuroimage* 47 756–763. 10.1016/j.neuroimage.2009.04.086 19426814

